# The Impact of Legal Recycling Constraints and Carbon Trading Mechanisms on Decision Making in Closed-Loop Supply Chain

**DOI:** 10.3390/ijerph19127400

**Published:** 2022-06-16

**Authors:** Yuyan Wang, Tingting Yu, Rui Zhou

**Affiliations:** 1 Business School, Shandong Normal University, Jinan 250014, China; 70008@sdnu.edu.cn; 2 School of Management Science and Engineering, Shandong University of Finance and Economics, Jinan 250014, China; 3 Department of Industrial and Systems Engineering, The University of Tennessee, Knoxville, TN 37996, USA; rzhou7@vols.utk.edu

**Keywords:** carbon emissions trading, recovery rate constraint, closed-loop supply chain, game theory

## Abstract

To investigate how legal constraints on the recycling rate of used products and carbon trading mechanisms affect the profits and other decisions of supply chain system members, this paper develops and solves a two-cycle game model in which the manufacturer dominates while the retailer takes a secondary position; the manufacturer produces only non-low-carbon new products in the first cycle and both new and low-carbon remanufactured products in the second cycle. Simultaneously, the effects of parameters such as recovery rate, unit carbon trading price, and carbon emission reduction factor on the decision making of members of the supply chain system are also discussed. Finally, the conclusions are verified by numerical analysis: (1) When carbon reduction is low, the manufacturer will choose the highest recycling rate to obtain the highest profit, and when carbon reduction is high, manufacturers tend to choose not to recycle when the minimum recycling rate bound by law harms the interests of manufacturers. (2) Under the implementation of the carbon trading mechanism, the unit carbon trading price affects the profits of the members of the supply chain system depending on the size of the carbon emission reduction, and the two are negatively correlated at lower carbon emission reductions and positively correlated at higher carbon emission reductions. (3) From the perspective of supply chain system members’ interests, legal constraints and the existence of carbon trading mechanisms are not always conducive to increasing the margins of members of the supply chain system, both relevant to the size of carbon emission reductions. (4) From the perspective of environmental benefits, supply chain members do not need to pay economic costs in all cases to contribute to environmental benefits, and the existence of minimum recycling rate constraints and carbon trading mechanisms are conducive to achieving carbon reduction targets.

## 1. Introduction

With the simultaneous growth of carbon emissions and total economic volume, side effects for the environment are gradually emerging, and adequately addressing global climate change and low-carbon economic development has become a hot topic worldwide [1]. In the face of many threats posed by the growth of carbon emissions, countries around the world have adopted legislation and declarations to respond to the development of a low-carbon economy, and major carbon-emitting countries, including China, have set timelines for achieving carbon neutrality goals [2]. In the process of achieving the carbon neutrality goal, various challenges will inevitably be faced. At present, global carbon reduction efforts have the following two difficulties that need to be solved urgently.

(1) The variety of waste products and the total amount is huge, and the recycling work is difficult. This is a common problem that countries around the world need to face. If not dealt with promptly, the harmful substances in the waste products will not only pose threats to the environment but also be detrimental to the realization of the overall goal of carbon emission reduction and carbon neutrality. For example, in the electronics industry, the total amount of e-waste is expanding annually at a rate of 3% to 5%, mainly because, as technology advances, the continuous upgrading of products has shortened the life cycle of electronic products. Faced with the same problem, the European Community has set a higher restriction on the recycling rate of used products, which has made some European countries succeed in e-waste disposal. In contrast, the production of electronic products in Africa is increasing, but the recycling situation is not optimistic. Asia and Latin America have also continuously introduced relevant laws and regulations to deal with the growing amount of e-waste year by year [3]. Some studies have pointed out that China may generate 27.22 million tons of electronic waste by 2030. In general, the treatment of electronic waste is a task that cannot be ignored [4]. For the disposal of electronic waste and other waste products, the most feasible way is to actively take effective ways and measures to recycle waste products and conduct low-carbon treatment, so that not only can the underutilized parts or materials in waste products be recycled, but environmental pollution caused by heavy metals such as lead, cadmium, chromium, and mercury can also be reduced.
(2) Low-carbon treatment of waste products after recycling is a challenging task. At the national level, because of the abundance of coal resources, the main energy consumption of China and Australia is still coal, which is a great challenge for the low-carbon transition of national energy [5]. For existing manufacturers, the implementation of the carbon trading mechanism has already increased their carbon reduction costs, resulting in a greater financial constraint, and the low-carbon treatment of waste products has put a heavier burden on them. It is very difficult for small and medium-sized manufacturers to make a comprehensive low-carbon transition. On the one hand, manufacturer enterprises of small and medium size are generally suffering from financial constraints, and a comprehensive low-carbon transformation requires high capital; moreover, the process of low-carbon transformation is subject to various risks, such as long lead times and mismatching of results with expectations, which can to a certain extent harm the economic interests of manufacturers. On the other hand, top managers play a pivotal role in the green transition. Compared with large manufacturers, top managers of small and medium-sized manufacturers have relatively weak awareness of low-carbon, environmental protection, and sustainable development, and are not inclined to join the low-carbon transition efforts from their subjective will [6]. These small and medium-sized manufacturer enterprises often need to rely on expensive external professional consultants to solve related problems at the request of their clients. Therefore, for manufacturers in the early stages of carbon reduction, it is currently feasible to reduce the environmental pollution and damage caused by used products by recycling used products and performing basic low-carbon treatment on them; for large-scale manufacturers with sufficient capital, a complete low-carbon transformation of product raw materials, production equipment, and processes is a desirable long-term plan. Siemens Infrastructure and Siemens Financial Services, a division of Siemens, announced in March 2022 that it would provide USD 100 million in ESG loans to U.S. SMEs, aimed at easing their decarbonization pressures. Within the scope of one’s ability, it is also a good choice to achieve a win–win situation by actively helping small and medium-sized manufacturer enterprises that have difficulties in reducing carbon emissions to complete the low-carbon transition.

However, the main research directions for waste recycling and carbon emission reduction currently focus on the conflict and governance of multiple recycling channels, the impact of recycling models on the profitability of supply chain members, and the comparison of carbon rights distribution methods [7,8,9,10]. The authors of these articles usually add some other factors, such as consumer environmental awareness, consumer preference, or competition among multiple homogeneous supply chain members, to the above-mentioned studies (relevant articles are described in detail in Section 2). There is not much coverage of the recycling of used products under legal constraints and carbon trading mechanism policies. The research in this paper takes into account the legal constraints on recycling rates and the joint impact of the implementation of the carbon trading system on optimal decision making and profits of two-cycle low-carbon closed-loop supply chain members, which is different from the existing papers. At the same time, this is also the knowledge gap and novelty of this paper. Consequently, are legal constraints on recycling rates necessary? How do legal constraints influence the profitability of each supply chain system member? How does the carbon trading mechanism affect the decision making of members of the supply chain system? How can the government act to most effectively achieve carbon emission reduction goals? We have developed a strong interest in this series of questions.

To effectively solve the above problems, based on the existing articles on single cycle production and remanufacturing activities [11,12,13,14,15], this paper refers to the model of articles [12,13,16], and a two-period Stackelberg game model is constructed in this paper with a single manufacturer as the dominant player and a single retailer as the follower. Since recycling of old products has actually become the choice of more and more manufacturers on the basis of low-carbon initiatives, the model established in this paper is more in line with the actual production process of manufacturer enterprises, and the conclusions drawn are more realistic and instructive. The following are the main contributions made by this paper:

(1) Most available articles are limited to examining restrictions on either the recycling rate or carbon trading price by law [17,18,19,20], or the comparison among different recycling channels and models as well as the advantages and disadvantages of different carbon rights allocation policies [21,22,23,24,25,26,27]. Different from most of the existing studies, this paper considers both the legal constraints on recycling rates and the carbon trading mechanism and studies their simultaneous effects on the decision making and profitability of members of the two-cycle closed-loop supply chain.
(2) In addition to analyzing the optimal decision-making problem from the perspective of supply chain members, this paper also puts forward policy suggestions from the perspective of government supervision. In the carbon emission reduction environment with a tight schedule and heavy tasks, it is not feasible to rely on the consciousness and responsibility of supply chain members alone, because in reality many manufacturing enterprises do not participate in recycling efforts for the sake of economic benefits, which makes carbon emissions seriously exceed the standard; moreover, the existence of a carbon trading market has also given rise to many gray industry chains. Therefore, how to effectively regulate the carbon emission behavior of manufacturers and maintain the stability of the carbon trading market is the key issue. As the lawmaker and main body regulating market order, the government is an objective factor that cannot be ignored in the decision-making process of manufacturers and retailers, and compared to existing research on the game between supply chain members, this paper studies from a tripartite perspective which has more practical significance.
(3) On the premise of studying the impact of legal constraints and the existence of carbon trading mechanisms on the decision making and profitability of members of the supply chain, this paper also analyzes the impact of carbon emission reduction, the influence coefficient of carbon emission reduction on the cost of remanufactured products, carbon trading price and carbon emission reduction coefficient on the profitability of the supply chain system. The conclusions supplement some of the existing studies, which not only provide a reference for the strategic choices that manufacturers and retailers should make based on different market environments, but also provides a research basis enabling the government to regulate the carbon emissions of manufacturers from different perspectives and regulate the order of the carbon trading market.

The rest of the chapters are arranged as follows. Section 2 outlines domestic and foreign studies related to the research of this paper. Section 3 depicts the problems studied by this paper. Section 4 constructs the research model and solves it. Section 5 discusses the relationship between the decision making and profitability of supply chain system members, the variations of each parameter, and compares the decisions and profits of members of the supply chain system with and without legal constraints. Section 6 provides numerical validation and analysis of the conclusions drawn from the model, and the article ends with the conclusions of the study in Section 7. The solution of the model and the proof of each proposition are attached in the Appendices.

## 2. Literature Review

Our research is associated with three research streams, namely the impact of legal constraints on recycling in the supply chain, the impact of carbon trading mechanisms on the supply chain, and the decision making of low-carbon closed-loop supply chains based on regulatory policy.

### 2.1. Impact of Legal Constraints on Recycling in the Supply Chain

As countries have become more concerned about environmental conditions, recycling has become one of the businesses that cannot be ignored by supply chain members. The existing literature on the subject focuses on the effect of legal constraints on recovery choices and margins in supply chain systems. By comparing three legal constraint models, i.e., no legal constraint, the legal constraint on recovery rate, and legal constraint on both recovery rate and recycling rate, Esenduran et al. discovered the effects of legal constraints on enterprises’ remanufacturing decisions, and the impact on environmental benefits, and finally found that if the law is too strict regarding recycling rate and remanufacturing rate, it would not be conducive to increasing manufacturer profits and the increase in recycling rate is not necessarily more conducive to the improvement of environmental benefits [20]. On this basis, Esenduran et al. found, by modeling original equipment manufacturers (OEMs) competing with independent recyclers (IRs), that stricter legal constraints on recovery rates do not imply an increase in remanufacturing rates, but OEMs benefit from stricter recycling legal constraints when OEMs reduce the amount of recycling and competitiveness of IRs [18]. Similarly, Wang et al. found that stricter legal constraints may lead to a worse situation for both economic and environmental benefits [28]. Building on this, Li et al. found that stricter recycling rates do not always harm manufacturers’ profits, but stricter recycling rate constraints always lead to lower profits; moreover, manufacturers’ optimal strategies are affected by potential recycling benefits [29]. Liu et al. confirmed that a stricter minimum collection rate constraint is generally detrimental to supply chain members when it becomes an active constraint [30]. In contrast to these studies, this paper specifies the conditions under which legal constraints harm the profits of supply chain members or not.

In addition, Mazahir et al. found through their study that setting a lower constraint level based on a single product characteristic is more conducive to improving environmental benefits than setting a uniform legal constraint level [31]. Hence, the government is required to set appropriate recycling constraint standards according to the different characteristics of different products instead of aiming for excessively high recycling targets. Otherwise, it is neither conducive to improving environmental benefits nor incentivizing manufacturers to design green products. Diabat and Jebali addressed the design of CLSC networks for durable products in conjunction with recycling legislation [32].

The above articles only consider the impact of legal constraints on recycling or remanufacturing rates on supply chain system members’ decisions and environmental benefits. They do not address the issue of carbon emissions in the production and remanufacturing processes of manufacturers. However, in reality, decisions in supply chain systems are often influenced not only by recovery rates but also by other factors. Therefore, unlike the above studies, based on the premise that all recycled products can be remanufactured, this paper adds an analysis of the carbon trading mechanism based on legal constraints on the recycling rate, which makes the study more relevant.

### 2.2. Impact of Carbon Trading Mechanism on Supply Chain Decision Making

The emergence of the carbon trading mechanism has brought more active carbon acquisition and carbon sales channels for manufacturers, and alleviated the economic losses suffered by manufacturers due to the inflexibility of the traditional fixed carbon quota mechanism. There are many research results on the effect of the carbon trading mechanism on the decision making of members of the supply chain system. Diabat et al. studied the impact of different carbon prices on the cost and structure of the supply chain by using numerical research methods [33]. Benjaafar et al. comparatively analyzed the impact of four low-carbon policies, namely, carbon trading system, carbon tax, carbon quota, and supply chain member cooperation, on supply chain production and carbon emission decisions [34]. Similarly, Fareeduddin et al. also compared and analyzed the cap and trade policy, carbon tax, and carbon cap policy in the article, and concluded that the cap and trade policy can well make up for the shortcomings of carbon tax and rigid carbon cap [35]. In addition, Mohammed et al. also believe that the carbon trading system is more flexible and effective than carbon offset and carbon cap policies [36]. Du studied how the cap-and-trading mechanism influences decision making and system performance in an emission-dependent supply chain [37]. The above studies all involve the impact of the carbon trading system on the supply chain, which lays a foundation for this paper to study the decision making of supply chain members based on the carbon trading system. However, different from the research conclusion of this paper, Xu investigated the optimal total carbon emissions of two products in a single manufacturer and single retailer supply chain, explored the impact of carbon trading price on the production choices of members of the supply chain, and found that both manufacturer’s and retailer’s profits showed a negative relationship with carbon trading price [38]. Turki et al. concluded that higher carbon trading prices are conducive to promoting recovery and remanufacturing [39]. Wang and Wu found the profits of supply chain members are negatively related to carbon trading price, and the profits of manufacturers are more sensitive to variations in the carbon trading price; overall, the trend of profit reduction has gradually slowed down, indicating that the impact of carbon trading prices has gradually declined [40]. Based on carbon trading, Wen et al. introduced consumer carbon awareness and investigated the emission reduction and pricing strategies of firms in a competitive market, influenced by the price of carbon trading and consumer carbon awareness, and the study found that higher carbon trading prices are not necessarily harmful to business interests; at the same time, for regulators in carbon emission industries aiming to reduce different levels of competition, increasing carbon prices is a better option than raising consumer awareness [41]. In contrast, the findings of Wen et al. are more consistent with those of this paper.

These existing studies have carefully discussed the impact of carbon trading mechanisms on supply chain members’ decision making and profits, but what changes will be made to the decision making of supply chain members when it comes to recycling used products and considering both legal recycling constraints and carbon trading mechanisms? Existing studies do not give a clear answer, which is the purpose of this study.

### 2.3. Decision Making of Low-Carbon Closed-Loop Supply Chains Based on Regulatory Policy

Supply chain members often aim at maximizing their interests when making decisions, while regulators hope to maximize social welfare and environmental benefits. Since the government’s intervention in production activities is one of the most effective ways to reduce environmental pollution [42], the decision making of supply chain members based on regulatory policies is worth exploring. In addition to earlier studies on carbon pricing issues [43], Heydari et al. studied the role of government regulation by enacting different types of government interventions, including tax exemptions and subsidies, and found that incentives for manufacturers are preferable to retailers [44]. Hafezalkotob considered the impact of six government intervention policies on the competition of green supply chains, and finally found that, compared with loose regulation, all government intervention policies are beneficial to the supply chain [45]. Mondal and Giri argue that both government subsidy and cap-and-trade policy are beneficial to all supply chain members [46]. On the contrary, Zand et al. found that direct government restrictions on the green level of products promote recycling of used products, increasing the profits of retailers but, to some extent, hurting the profits of manufacturers [47]. Wang et al. explored recycling decisions of the closed-loop supply chain of low-carbon e-commerce on the basis of the effect of the government subsidy mechanism and altruistic preference, and the study pointed out that government subsidies increased the operational efficiency and total social welfare of the whole supply chain [48]. Zhang et al. explored the optimal decision of a two-channel supply chain under four reward–penalty policies (RPP), i.e., no policy constraint, carbon emission constraint only, recycling rate constraint only, and both carbon emission and recycling rate constraint, and concluded that the three constrained government RPPs were effective in controlling total carbon emissions and benefiting social welfare, though in most cases to the detriment of the interests of retailers [49]. Based on this, Chen et al. proposed that manufacturers should be regulated for both carbon emissions and recycling rate, because these two regulations play an important role in the achievement of the dual objectives of carbon reduction and resource recovery and are complementary [50]. Zhang and Li explored the impact of corporate social responsibility (CSR) on dual-channel closed-loop supply chain performance based on carbon tax or subsidy policies and found that carbon tax policies were applied to supply chains that implemented CSR activities, while subsidy policies were applied to supply chains that did not implement CSR activities [51]. Luo et al. assessed the implications of carbon tax policies on low carbon supply chain manufacturing and remanufacturing strategies [52].

The above articles all focus on the impact of either the legally binding recycling rate or the carbon trading mechanism on the optimal decision of members of the supply chain system. The difference of this paper is that it integrates legal recycling constraints and the carbon trading mechanism to explore the influence of the two on the decision making of the low-carbon closed-loop supply chain, gains insights from the research results, and provides constructive suggestions.

The differences between this article and some articles are shown in Table 1.

## 3. Problem Statement

**Figure 1:**
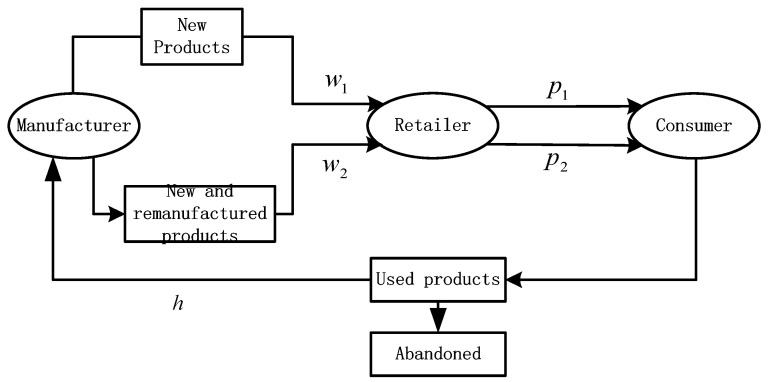
Model structure diagram.

The model considers a closed-loop supply chain system involving a manufacturer and a retailer, as shown in Figure 1. In this closed-loop supply chain, the manufacturer is accountable for producing both new and remanufactured products and recycling used products; the retailer is accountable for selling new and remanufactured products. For the purpose of analyzing how legal constraints and the carbon trading mechanism affect the recycling of used products, the model considers the operational activities of two production cycles before and after the implementation of the carbon trading mechanism, and the life cycle of new products and remanufactured products is only one cycle [54]. In the first cycle, without the implementation of a carbon trading mechanism, the manufacturer only produces new products; when the life cycle of the products ends, the manufacturer collects used products from the waste recycling market following the legal constraints on the number of used products to be recycled. Subsequently, the manufacturer wholesales the non-low-carbon products at a certain wholesale price to the retailer, who then sells them to the consumer market. In the second cycle, the policy begins to implement the carbon trading mechanism. In addition to producing new products, the manufacturer processes used products recovered in the first cycle and sells both new and remanufactured products to the retailer, who sells them to the market to meet consumer demand at the same wholesale price [44,55,56,57].

Under the implementation of the carbon trading mechanism, in the second production cycle, the government determines the initial carbon allowances based on the total carbon emissions of the previous cycle and allocates them to the manufacturer to ease the pressure on carbon emissions [58]. When the carbon emissions per unit cycle of products are less than the free carbon allowances, the surplus carbon allowances are profitable when sold into the carbon trading market; however, when the carbon emissions per unit cycle of products are greater than the free carbon allowances, the manufacturer is compelled to purchase carbon allowances from the carbon trading market to meet its carbon demand [53]. Under the constraint of the carbon trading mechanism, the manufacturer will adopt lower costs to achieve the lowest possible carbon emissions. Since the production process, management, and equipment of new products have been built in the first cycle, it is more costly to adjust to the low carbon process; however, the remanufacturing processing of used products is different. In the second cycle, the processing of remanufactured products has just started, so the manufacturer will adopt advanced low carbon production processes for the remanufacturing of used products to ensure the carbon emissions from remanufactured products are smaller than those from new products so as to improve its carbon revenue which also causes remanufacturing work to cost more than producing new products.

The variables and parameters addressed by the model are shown in Table 2.

To ensure that the model makes sense, the assumptions are as follows:

(1) In the supply chain model, the manufacturer is assumed to be the core dominant firm and the retailer is the subordinate firm.
(2) Assumption that the manufacturer conducts rigorous testing of the used products recycled after the first cycle to ensure that the recycled used products have a certain utilization value and can be processed into remanufactured products in the second cycle [59].
(3) Drawing on the assumptions of the literature [44,55,56,57], the remanufactured products are of the same quality as new products, and are sold to the market at the same price to meet consumer demand. For example, the remanufacturing of products such as glass bottles and jars does not result in any loss of quality.
(4) Assumption that the processing of remanufactured products adopts a low-carbon process, which makes the carbon emissions of remanufactured products smaller than those of new products, i.e., $e>e_{0}$; it also results in the remanufactured products costing more to produce than new products, drawing on the assumptions of the literature [60]. The processing cost of remanufactured products is assumed to be proportional to carbon emission reduction savings s ($s=e-e_{0}$), i.e., $c_{0}=\mu +k(e-e_{0})$.
(5) Since the recycling rate of the product may not actually reach 100% [61] (for example, many people who once used old cell phones do not want them to be recycled because they are worried about safety), it is assumed that the maximum possible recycling rate of the waste product is $h_{1}$ ($h_{1}\leq 1$), thus the actual recycling rate of used products needs to meet $h_{1}\geq h\geq h_{0}$.
(6) Remanufactured products cannot fully meet consumer demand for products, so, in the second cycle, the manufacturer not only processes remanufactured products but also produces new products, i.e., $hq_{1}<q_{2}$.
(7) Free carbon allowances for the current period are allocated through a grandfathering system, that is, the initial amount of carbon allowances is based on the total carbon emissions of the previous period [62]. The manufacturer receives free carbon allowances for a single cycle only and these cannot be carried over to the next cycle.

According to the above statement of assumptions, the manufacturer’s profit function is:

(1) $$\pi_{m}=(w_{1}-c)q_{1}+(w_{2}-c)(q_{2}-hq_{1})+(w_{2}-c_{0})hq_{1}+[\theta eq_{1}-e(q_{2}-hq_{1})-e_{0}hq_{1}]P_{C}$$

In Equation (1), the first term represents profit from the wholesale of the new product in the first cycle. The second and third terms represent profit from the wholesale of new and remanufactured products in the second cycle, respectively, and the fourth term is the transaction cost or revenue of carbon allowances in the second cycle, where the free carbon allowances allocated by the government are equal to the carbon emission reduction coefficient multiplied by the total carbon emissions in the first cycle, i.e., $\theta eq_{1}$.

The retailer’s profit function is:

(2) $$\pi_{r}=(p_{1}-w_{1})q_{1}+(p_{2}-w_{2})q_{2}$$

In Equation (2), the first term represents the profit from selling new products in the first cycle, and the second term represents the profit from selling products (both new and remanufactured products) in the second cycle.

The profit of the supply chain system is:

(3) $$\pi =\pi_{m}+\pi_{r}$$

## 4. Model Construction and Solution

### 4.1. Model without Considering Legal Recovery Constraints (F-Model)

Without considering legal recycling constraints, the manufacturer’s decision function is:

(4) $$\begin{array}{c} \underset{w_{1},w_{2},h}{\max}\pi_{m}=(w_{1}-c)q_{1}+(w_{2}-c)(q_{2}-hq_{1})+(w_{2}-c_{0})hq_{1}+[\theta eq_{1}-e(q_{2}-hq_{1})-e_{0}hq_{1}]P_{C}\\ s.t.\;0\leq h\leq h_{1} \end{array}$$

The retailer’s decision function is:

(5) $$\underset{p_{1},p_{2}}{\max\;}\pi_{r}=(p_{1}-w_{1})q_{1}+(p_{2}-w_{2})q_{2}$$

In the model, a Stackelberg game consists of a manufacturer and a retailer, with the manufacturer being the dominant player and the retailer being the subordinate player. In decision making, the manufacturer takes the lead in determining the wholesale prices $w_{1},w_{2}$ and the recovery rate h; the retailer then decides the market selling prices $p_{1}$ and $p_{2}$ of products based on the manufacturer’s decisions. Using the inverse induction method, the specific solution process is shown in Appendix A and the optimal solution obtained by solving the model is shown in Table 3 (the superscripts F*,L* represent the optimal decision of the F-model and L-model, respectively).

The optimal decisions in Table 3 show that the manufacturer’s optimal recycling rate varies with the value of s ($s=e-e_{0}$).

The optimal recycling rate h of the manufacturer without the legal recycling constraint is zero when the carbon saving s is large ($s>s_{1}$) because the manufacturer’s production cost $c_{0}$ ($c_{0}=\mu +ks$) for processing a unit of remanufactured product is higher when the carbon saving is large compared to processing a new product, resulting in a higher profit for a new product than a remanufactured product. Thus, the manufacturer tends to choose not to recycle when the legal recycling constraint is not considered.

The optimal recycling rate of the manufacturer without the legal recycling constraint is maximized when the carbon saving s is small ($s<s_{1}$). Smaller carbon saving results in lower production cost $c_{0}$ for the manufacturer to process a unit of remanufactured product. It is more profitable to process a remanufactured product than to produce a new product, so the manufacturer makes the greatest effort to recycle used products resulting in the greatest recycling rate.

The optimal decisions in Table 3 show that $w_{2}$ and $p_{2}$ of the products in the second cycle are not affected by changes in the values of s and recovery rate h. This is mainly because, in the second cycle, the market price of new products and remanufactured products is the same and there is no difference in the consumer experience that both bring to consumers in terms of quality and performance. Additionally, it is twice as profitable for the manufacturer as it is for the retailer, indicating that the manufacturer takes a dominant position in the market and can use the dominant power to obtain more benefits.

### 4.2. Model with Legal Recovery Constraints (L-Model)

Under the legal recycling constraints and carbon trading mechanism, the manufacturer’s decision function is:

(6) $$\begin{array}{c} \underset{w_{1},w_{2},h}{\max}\pi_{m}=(w_{1}-c)q_{1}+(w_{2}-c)(q_{2}-hq_{1})+(w_{2}-c_{0})hq_{1}+[\theta eq_{1}-e(q_{2}-hq_{1})-e_{0}hq_{1}]P_{C}\\ s.t.h_{0}\leq h\leq h_{1} \end{array}$$

The retailer’s decision function is:

(7) $$\underset{p_{1},p_{2}}{\max\;}\pi_{r}=(p_{1}-w_{1})q_{1}+(p_{2}-w_{2})q_{2}$$

The optimal solution for solving this model is shown in Table 3.

As shown in Table 3, when the legal recycling constraint is considered and when the carbon saving s is large ($s>s_{1}$), the production cost of remanufactured products processed by the manufacturer is higher than that of new products, which means that the profit obtained by producing new products will be greater than that of producing remanufactured products. Thus, the manufacturer’s optimal recycling rate h is the legally constrained minimum recovery rate $h_{0}$. When the saving s is small ($s<s_{1}$), the manufacturer’s optimal recovery rate is $h_{1}$, which is consistent with the case where legal recycling constraint is not considered.

## 5. Model Comparison and Analysis

A comparative analysis of the optimal decisions with and without the legal recovery constraints model leads to the following conclusions:

**Proposition** **1.** (1) $w_{{}_{1}}^{i},p_{{}_{1}}^{i}(i=F*,L*)$ are positively correlated with k and negatively correlated with $e_{0}$; (2) $q_{{}_{1}}^{i}(i=F*,L*)$ is negatively correlated with k and positively correlated with $e_{0}$; (3) $w_{{}_{2}}^{i},p_{{}_{2}}^{i},q_{{}_{2}}^{i}(i=F*,L*)$ are not affected by the values of k and $e_{0}$; (4) $\pi_{m}^{i},\pi_{r}^{i},\pi^{i},(i=F*,L*)$ are negatively correlated with k and positively correlated with $e_{0}$.

Proofs see Appendix B.

Proposition 1 reveals that:

(1) $w_{{}_{1}}^{i},p_{{}_{1}}^{i}(i=F*,L*)$ increase as the cost influence coefficient k increases, market demand $q_{{}_{1}}^{i}(i=F*,L*)$ decreases as the cost influence coefficient k increases, and the manufacturer, the retailer, and the supply chain system profits $\pi_{m}^{i},\pi_{r}^{i},\pi^{i},(i=F*,L*)$ decrease as the cost influence coefficient k increases.

The cost influence coefficient k indicates the degree of impact of the change in carbon emission reduction on the cost of remanufactured products and k is positively correlated with $c_{0}$. When the value of k is larger, the cost of remanufactured products $c_{0}$ is higher. In order to obtain a certain product profit, the manufacturer will raise the wholesale price $w_{1}$, and the retailer will also raise the retail price $p_{1}$ due to the increase in wholesale price resulting in lower market demand $q_{1}$ for products. The profits of supply chain system members are negatively correlated with k, which means that, on the one hand, as the cost influence coefficient increases, the profits gained by the manufacturer and the retailer by increasing price are less than the loss caused by the reduction of market demand due to price increases; on the other hand, the larger the cost influence coefficient k is, the more cost will be invested in the low-carbon process of processing remanufactured products, and therefore the profit per unit of product will decrease. It also indicates that in the process of production and operation, manufacturer enterprises need to actively develop and innovate low-carbon production processes and form a set of low-carbon production processes suitable for themselves, so as to achieve a net increase in their profits based on reducing process costs.

(2) $w_{{}_{1}}^{i},p_{{}_{1}}^{i}(i=F*,L*)$ decrease with increasing carbon emissions of remanufactured products $e_{0}$, $q_{{}_{1}}^{i}(i=F*,L*)$ increases with increasing carbon emissions of remanufactured products $e_{0}$, and $\pi_{m}^{i},\pi_{r}^{i},\pi^{i},(i=F*,L*)$ increase with increasing carbon emissions of remanufactured products $e_{0}$.

The carbon emission per unit of remanufactured product $e_{0}$ is negatively correlated with $c_{0}$. When the value of $e_{0}$ is larger, the $c_{0}$ is lower, and the manufacturer and the retailer will reduce wholesaling and retailing prices of products. Thus, the demand for products in the market will increase. $\pi_{m}^{i},\pi_{r}^{i},\pi^{i},(i=F*,L*)$ increase with increases in $e_{0}$, which indicates that the strategy of the manufacturer and the retailer to sell more productions at a lower price is effective, promoting the sale of products and gaining more profits at the same time. It is also because of the positive correlation between $e_{0}$ and $\pi_{m}^{i},\pi_{r}^{i},\pi^{i},(i=F*,L*)$ that some manufacturer enterprises will not pay attention to environmental benefits for the sake of economic benefits, making the carbon emissions of remanufactured products increase, which is not conducive to the accomplishment of carbon reduction goals and the realization of manufacturers’ social values. Therefore, it is necessary for the law to force manufacturer enterprises to carry out recycling efforts through a minimum recycling rate constraint in order to reduce the carbon emissions generated by manufacturers’ production activities.

(3) $w_{{}_{2}}^{i},p_{{}_{2}}^{i},q_{{}_{2}}^{i}(i=F*,L*)$ are not affected by the remanufactured product cost influence coefficient k and the carbon emission per unit of remanufactured product $e_{0}$, but are related to the total production cost per unit of the new product which equals the sum of the production cost and carbon emission cost per unit of the new product. This is due to the manufacturer producing both new and remanufactured products which will be sold by the retailer at the same price in the second cycle. If the second-cycle price of products is determined based on the cost of remanufactured products $c_{0}$ (where $c_{0}=\mu +k(e-e_{0})>c$), the products will be sold at a higher price than new products in the first cycle. In the long run, prices will gradually increase, which will not only affect consumers’ consumption experience but also break the equilibrium state of the market.

**Proposition** **2.** (1) $w_{{}_{1}}^{i},p_{{}_{1}}^{i}(i=F*,L*)$ are negatively correlated with unit carbon allowance trading price $P_{C}$ and carbon emission reduction coefficient θ, respectively; (2) $w_{{}_{2}}^{i},p_{{}_{2}}^{i}(i=F*,L*)$ are positively correlated with $P_{C}$ and not affected by θ; (3) $q_{{}_{1}}^{i}(i=F*,L*)$ is positively correlated with $P_{C}$ and θ, respectively, and $q_{{}_{2}}^{i}(i=F*,L*)$ is negatively correlated with $P_{C}$ and not affected by θ; (4) $\pi_{m}^{i},\pi_{r}^{i},\pi^{i},(i=F*,L*)$ are positively correlated with θ, when $s>s_{2}$ positively correlated with $P_{C}$, and when $s<s_{2}$ negatively correlated with $P_{C}$.

**Proof.**  The same idea as Proposition 1 (where $s_{2}=\frac{e(1-\theta )}{h}$). □

Proposition 2 reveals that:

(1) $w_{{}_{1}}^{i},p_{{}_{1}}^{i}(i=F,L)$ decrease as the unit carbon allowance trading price $P_{C}$ increases, and $q_{{}_{1}}^{i}(i=F,L)$ increases as the value of $P_{C}$ increases. This is because the carbon allowances obtained by the manufacturer in the second cycle are related to the production of products in the first cycle, and as $P_{C}$ increases the manufacturer tries to make the demand for the products in the first cycle greater to obtain more carbon trading revenue; therefore, as $P_{C}$ increases, the manufacturer joins with the retailer to lower prices, thus increasing the demand for their products.

$w_{{}_{2}}^{i},p_{{}_{2}}^{i}(i=F,L)$ increase with the increase in $P_{C}$, and $q_{{}_{2}}^{i}(i=F,L)$ decreases with the increase in $P_{C}$. This is because in the second cycle, with the increase in carbon allowance trading price, manufacturer enterprises will be divided into two categories. One category will try to reduce the carbon emissions of remanufactured products to achieve maximum carbon savings and then profit from selling additional carbon allowances. In this case, the cost of investing in carbon reduction processes will increase. The other category of manufacturers need to purchase additional carbon allowances to complete their production because the carbon reduction process is not perfect and the existing carbon allowances are not enough to meet production demand, which will also increase the production cost of manufacturer enterprises. Regardless of whether the carbon allowance is sufficient or not, the cost of remanufactured products will increase, so the members of the supply chain system will increase the selling price of products and market demand will decrease in the end.

The profits $\pi_{m}^{i},\pi_{r}^{i},\pi^{i},(i=F,L)$ show a trend of decreasing and then increasing with increases in unit carbon trading price $P_{C}$. That is, when $s<s_{2}$, the profits of members of the supply chain system decrease with the increase in $P_{C}$, and when $s>s_{2}$ the profits of members of the supply chain system increase with increases in $P_{C}$. These indicate that when the value of s is low, with increases in $P_{C}$, the profit gained by selling the excess carbon allowances is less than the cost invested in the low carbon process, or that the manufacturer does not have enough carbon allowances and needs to spend a lot of money to buy additional carbon allowances in order to meet production, which further increases production cost and reduces the profits of supply chain members. On the contrary, when the value of s is high and because the low-carbon production process is more mature, as the price of carbon trading increases, the manufacturer can earn significant revenue by selling excess carbon allowances, which is greater than the sum of the cost invested in the low-carbon process and the loss of reduced market demand due to price increases.

(2) $w_{{}_{1}}^{i},p_{{}_{1}}^{i}(i=F,L)$ decrease with increase in θ, and $q_{{}_{1}}^{i}(i=F,L)$ increases with increase in θ. Because when θ is larger the manufacturer gets higher carbon allowances $\theta eq_{1}$, in the second cycle, in order to be able to sell the excess carbon allowances in the second cycle to get more carbon trading revenue, the manufacturer will reduce the product price in the first cycle jointly with the retailer to promote product sales.

$w_{{}_{2}}^{i},p_{{}_{2}}^{i},q_{{}_{2}}^{i}(i=F,L)$ are not affected by θ. The carbon emissions reduction coefficient θ depends on the overall level of the industry and has minimal impact on the production of individual supply chain companies. However, $\pi_{m}^{i},\pi_{r}^{i},\pi^{i},(i=F,L)$ increase as the carbon emission reduction coefficient θ increases. This indicates that the higher market demand due to lower prices results in a more lucrative profit for the manufacturer, which is higher than the loss due to price reduction, as shown by the increase in profits obtained by supply chain members with increases in θ.

The size relationships comparing the optimal decisions of the F-model and L-model are shown in Table 4.

By observing Table 4, we can see that:

(1) When the value of s is high ($s>s_{1}$), the wholesale price and retail price of new products in the first cycle are lower and market demand is higher when legal recycling constraints are not considered, i.e., $w_{1}^{F*}<w_{1}^{L*}$, $p_{1}^{F*}<p_{1}^{L*}$, $q_{1}^{F*}>q_{1}^{L*}$.This is because when s is higher, the cost of the manufacturer’s investment in carbon emission reduction equipment and the process is also higher. Compared to the case where legal constraints are considered, the manufacturer will choose not to do the recycling work when legal constraints are not considered, so the manufacturer is not required to pay the high cost of remanufacturing the used products; therefore, the market demand for products can be increased by reducing the price of the product. Under the two conditions with or without legal constraints, there is no difference in $w_{{}_{2}}^{i},p_{{}_{2}}^{i}(i=F*,L*)$ or the market demand for products in the second cycle. This is because the price of the products in the second cycle relates only to the production cost of the new products and the cost of carbon emission, which are all determined after the production process and equipment are built in the first cycle. Thus, the decision variables in the second cycle are not affected by legal constraints. By comparing the magnitude of profits, we find that $\pi_{m}^{F*}>\pi_{m}^{L*}$, $\pi_{r}^{F*}>\pi_{r}^{L*}$, and $\pi^{F*}>\pi^{L*}$, i.e., the profits of the members of the supply chain system and the total profits of the supply chain system under the legal recycling constraint are lower than those without the legal constraint, which indicates that the legal constraint set to facilitate the development of low-carbon production by promoting the reduction of carbon emissions affects the economic interests of each member of the supply chain to some extent.

Under the circumstance that the interests of members of the supply chain system will be damaged when carbon emission reduction is promoted, to cooperate with legal constraints and promote manufacturer enterprises to carry out recycling and remanufacturing work, the government can provide incentives to manufacturer enterprises by giving certain remanufacturing subsidies, rewarding benchmark manufacturers with excellent remanufacturing work, establishing a low-carbon production process fund, or organizing related innovation activities. On the other hand, manufacturer enterprises can reduce carbon emissions in the production process by striving to develop environmentally-friendly low-carbon production processes and gain higher profits by reducing carbon emission costs.

(2) When the value of s is low ($s<s_{1}$), the profit of remanufacturing a used product is higher than processing a new product, and the manufacturer is motivated to choose the maximum recovery rate to remanufacture used products. At this time, the minimum recovery rate stipulated by law has little effect. In other words, without legal constraints, the manufacturer will actively engage in recycling to maximize not only its interests but also the interests of the entire supply chain system. In this case, the minimum recycling rate stipulated by law is weak for large manufacturer enterprises with perfect carbon reduction processes; however, for some small and medium-sized manufacturer enterprises with insufficient green recycling conditions, the law can appropriately increase the minimum recycling rate to regulate their recycling of used products in order to respond to the national policy of a low carbon and circular economy, which is a more ideal situation for all parties to win.

## 6. Numerical Analysis and Discussion

### 6.1. Numerical Analysis

Ten years ago, the EU stipulated that the recovery rate of e-waste should reach 45% and hoped that the recovery rate of e-waste would reach 65% by 2019 [63]. Currently, the EU hopes to achieve a plastic recycling rate of 55% by 2023. However, the plastic industry has problems of high waste rates, large amount of required recycling, and difficulty of the recycling process. Compared to other waste products, such as electronic products, the plastic recycling rate is often relatively low. Moreover, the average recovery rate of electric vehicle batteries in EU member states at this stage is 48% [64]. It can be seen that the difficulty of recycling different products is different. Therefore, the minimum recovery rate of 0.55 is taken in this paper to represent the recovery rate of waste products in e-waste, plastics, and other industries with relatively high recovery rates.

One of the technical challenges set by the UK Institute is to achieve a 95% recycling rate for EV battery packs by 2035 [64], which is an unrealized goal. Because the waste products, in reality, can not be completely recycled, the maximum recycling rate set in this paper is 0.9.

The conclusions of this paper are further analyzed in-depth by the theoretical values below.

**Figure 2:**
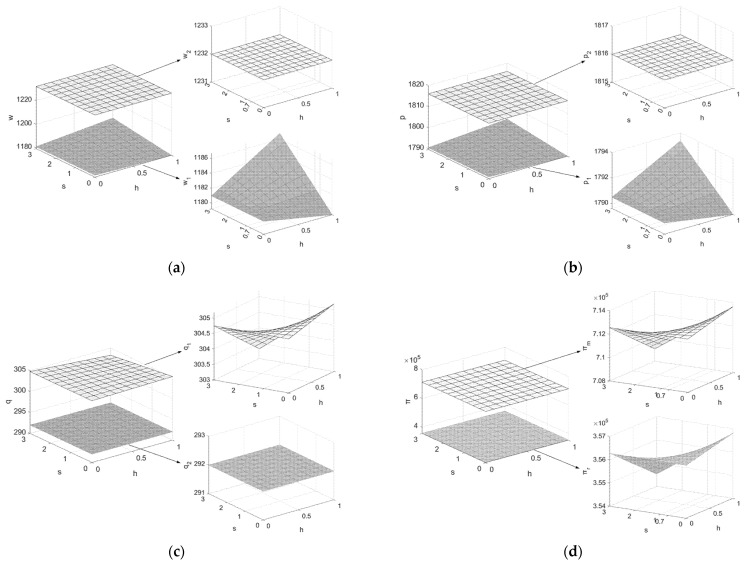
Effects of h and s on decision variables. (**a**) Effects of h and s on $w_{1},w_{2}$; (**b**) effects of h and s on $p_{1},p_{2}$; (**c**) effects of h and s on $q_{1},q_{2}$; (**d**) effects of h and s on $\pi_{m},\pi_{r},\pi$.

1. Assuming Q=1200, c=4, μ=0.5, e=3, $h_{0}=0.55$, $h_{1}=0.9$, $\beta_{1}=\beta_{2}=0.5$, k=25, θ=0.7, $P_{C}=20$, letting h and s be the independent variables (h∈[0,1], s∈[0,3], at this point $s_{1}=\frac{c-\mu }{k-P_{C}}=0.7$), draw graphs of the variation of the decision variables and system members’ profits with h and s, as shown in Figure 2.

From Figure 2: (1) When the value of s is below the threshold value $s_{1}$, the carbon emission of remanufactured products is higher and increasing the recycling rate cannot bring the manufacturer higher profits from selling carbon rights. However, it will increase the manufacturer’s recycling workload, so the manufacturer tends not to do recycling work, i.e., the recycling rate chosen by the manufacturer is zero without legal constraints and $h_{0}$ with legal constraints. Additionally, the manufacturer will choose to lower the price of new products in the first cycle to stimulate demand in order to obtain higher carbon allowances in the second cycle. When s is higher than $s_{1}$, increasing the recycling rate can not only achieve the purpose of energy saving and emission reduction, but also can increase the manufacturer’s carbon trading revenue. Thus, supply chain members do not need to choose the strategy of selling more products at a lower price to obtain a higher carbon quota. At the same time, due to the higher cost of investing in the emission reduction process in the second cycle, the manufacturer and the retailer choose to increase the selling price of their products to gain a certain profit. (2) When the value of s is lower than the threshold value $s_{1}$, the profits of supply chain system members are positively correlated with the recycling rate h, which means that the manufacturer will choose the highest recycling rate $h_{1}$ to maximize profit, and the main function of the minimum recycling rate stipulated by law is to restrain the small and medium-sized manufacturer enterprises who are backward in terms of emission reduction. Conversely, when s is higher than $s_{1}$, the profits of system members are negatively correlated with h, and the manufacturer will choose not to recycle as much as possible to ensure profit is not damaged. To avoid the situation where the manufacturer’s recycling rate is zero, which is not conducive to achieving the carbon emission reduction target, it is necessary for the law to constrain the minimum recycling rate. At the same time, the law should also constrain the carbon emission reduction of remanufactured products, while constraining the recycling rate of used products, so as to avoid the situation where the recovery rate of used products determined by the manufacturer meets the standard but the carbon emission reduction per unit of remanufactured product is low, which ultimately fails to achieve the carbon emission reduction targets.

**Figure 3:**
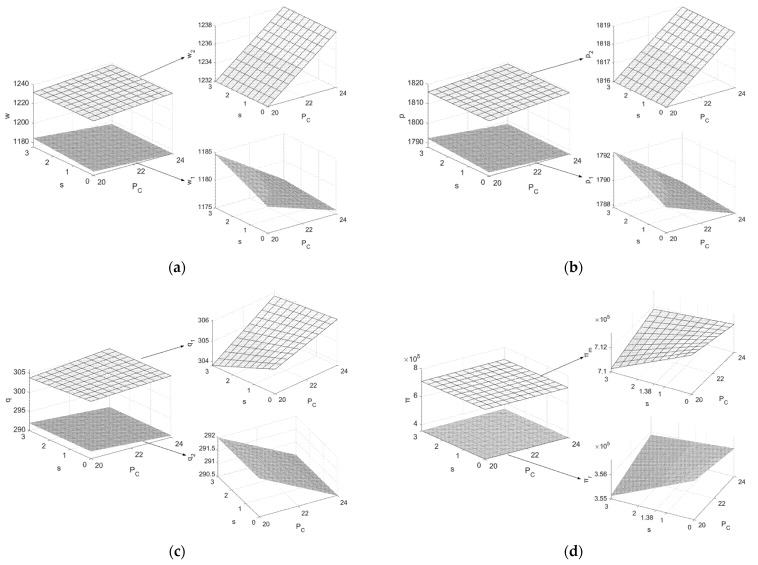
Effects of $P_{C}$ and s on decision variables. (**a**) Effects of $P_{C}$ and s on $w_{1},w_{2}$; (**b**) effects of $P_{C}$ and s on $p_{1},p_{2}$; (**c**) effects of $P_{C}$ and s on $q_{1},q_{2}$; (**d**) effects of $P_{C}$ and s on $\pi_{m},\pi_{r},\pi$.

2. Assuming Q=1200, c=4, μ=0.5, e=3, $h_{0}=0.55$, $h_{1}=0.9$, $\beta_{1}=\beta_{2}=0.5$, k=25, h=0.65, θ=0.7, letting $P_{C}$ and s be the independent variables ($P_{C}\in [20,24]$, s∈[0,3], at this point $s_{2}=1.38$), draw graphs of the variation of the decision variables and system members’ profits with $P_{C}$ and s, as shown in Figure 3.

From Figure 3: (1) When the unit carbon trading cost $P_{C}$ is high, to obtain more free carbon allowances in the second cycle the manufacturer will reduce $w_{{}_{1}}^{i},p_{{}_{1}}^{i}(i=F*,L*)$ in the first cycle to stimulate demand. When the carbon emission reduction s is larger ($e_{0}$ is smaller), the desire of the manufacturer to benefit from selling additional carbon allowances at a high price is greater, which further motivates the manufacturer to want to obtain high carbon allowances, which is manifested as a larger price reduction in the first cycle. In the second cycle, the positive relationship between $w_{{}_{2}}^{i},p_{{}_{2}}^{i}(i=F*,L*)$ and $P_{C}$ indicates that the manufacturer chooses to raise the wholesale price in the second cycle to get some profit as the cost of carbon trading increases. In order to keep the market stable, the government needs to regulate the carbon trading price to fluctuate within a certain range. (2) With increase in the unit carbon trading cost $P_{C}$, the trend of profits gained by the supply chain system members is related to the value of s. When $s<s_{2}$ ($s_{2}=1.38$), the profits of system members show a decreasing trend. The smaller s is, the larger the profit reduction is, which indicates that the profit loss caused by the increase in carbon trading price is greater for manufacturer enterprises with poor carbon emission reduction processes than for manufacturer enterprises with good carbon emission reduction processes. Conversely, with increases in s, when $s>s_{2}$ the profits of system members show an increasing trend, and the larger the value of s, the faster the increase in the profits of system members. This indicates that the better the carbon reduction process is, the higher the carbon trading profit the manufacturer receives. Therefore, an appropriate increase in unit carbon trading price is conducive to motivating the manufacturer to actively transform and upgrade low-carbon processes in order to obtain higher profits.

Furthermore, system members are most profitable when both $P_{C}$ and s are at their minimum values. Therefore, when $P_{C}$ is at a minimum, the manufacturer is likely to stop working on reducing carbon emissions of remanufactured products and will sacrifice the environment to achieve the highest profit. To avoid this extreme situation, on the basis of balancing $P_{C}$, the government also needs to restrain the carbon emission per unit of remanufactured product produced by manufacturers, give corresponding policy incentives to manufacturer enterprises with excellent carbon reduction, and punish those enterprises who are lagging in terms of carbon reduction efforts, subsequently promoting the smooth operation of low-carbon recycling of used products by restraining the recycling rate of each manufacturer. From the manufacturer enterprises’ point of view, it is not long-term to pursue high profits and ignore environmental benefits in production and operation activities. Only by actively taking social responsibility can they get more support from regulators and consumers and sustain development in a good environment.

**Figure 4:**
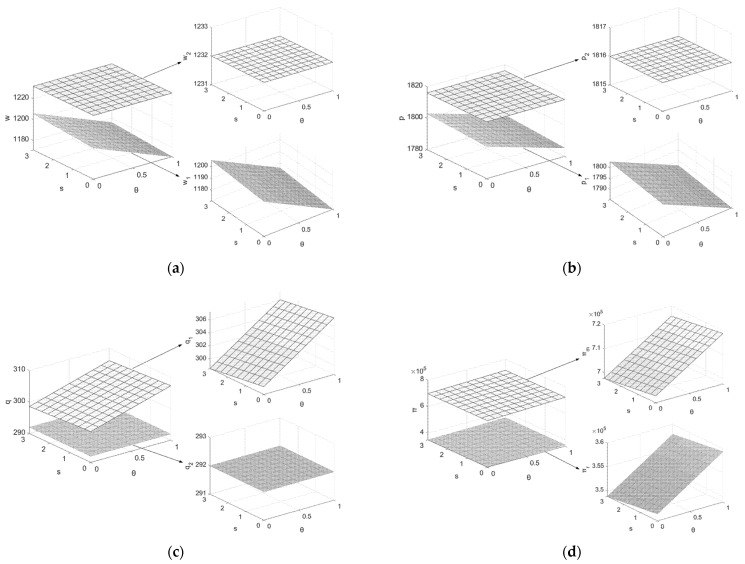
Effects of θ and s on decision variables. (**a**) Effects of θ and s on $w_{1},w_{2}$; (**b**) effects of θ and s on $p_{1},p_{2}$; (**c**) effects of θ and s on $q_{1},q_{2}$; (**d**) effects of θ and s on $\pi_{m},\pi_{r},\pi$.

3. Assuming Q=1200, c=4, μ=0.5, e=3, $h_{0}=0.55$, $h_{1}=0.9$, $\beta_{1}=\beta_{2}=0.5$, k=25, $P_{C}=20$, h=0.65, letting θ and s be the independent variables (θ∈(0,1], s∈[0,3], at this point $s_{1}=\frac{c-\mu }{k-P_{C}}=0.7$), draw graphs of the variation of the decision variables and system members’ profits with θ and s, as shown in Figure 4.

From Figure 4, (1) $w_{{}_{1}}^{i},p_{{}_{1}}^{i}(i=F*,L*)$ decrease as θ increases and $q_{{}_{1}}^{i}(i=F*,L*)$ increases as θ increases. This indicates that higher θ provides higher carbon quotas to the manufacturer for the second cycle of production activities, and the manufacturer does not need to spend more money on carbon trading. Therefore, while the supply chain system members attract consumers through a thin profit strategy to gain sales profits, the increase in demand for new products in the first cycle is more favorable for obtaining higher carbon quotas in the second cycle. (2) $\pi_{m}^{i},\pi_{r}^{i},\pi^{i},(i=F*,L*)$ increase with θ, which indicates that additional carbon allowances in the second cycle provide higher economic profits for system members. However, when s is the smallest and θ is the highest, the supply chain member companies can obtain the maximum profit. To avoid some manufacturer enterprises from not making efforts to reduce carbon emissions to maximize their profits, it requires the government to reasonably regulate the size of the carbon emission reduction coefficient and set a minimum limit for carbon emission reduction per unit of remanufactured product. In short, too high a value of θ is not conducive to achieving carbon reduction targets, while too low a value of θ is detrimental to the interests of system members, which is a long-term game between the government and supply chain members.

**Figure 5:**
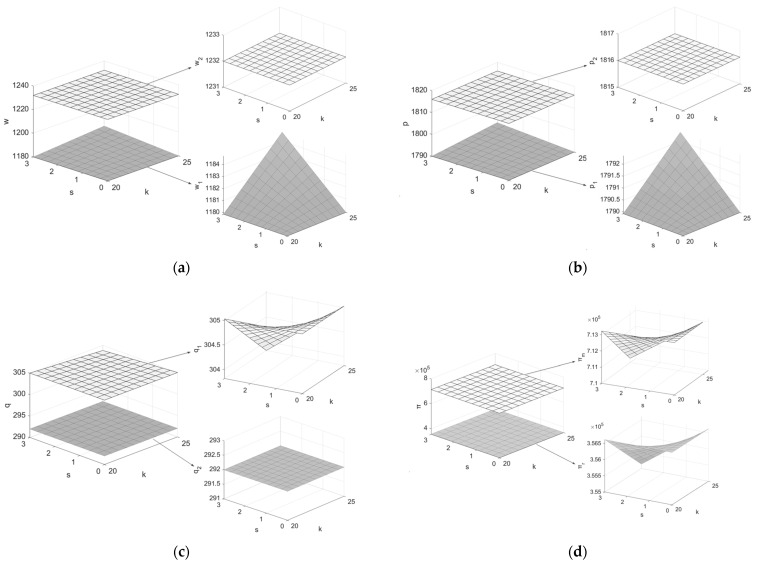
Effects of k and s on decision variables. (**a**) Effects of k and s on $w_{1},w_{2}$; (**b**) effects of k and s on $p_{1},p_{2}$; (**c**) effects of k and s on $q_{1},q_{2}$; (**d**) effects of k and s on $\pi_{m},\pi_{r},\pi$.

4. Assuming Q=1200, c=4, μ=0.5, e=3, $h_{0}=0.55$, $h_{1}=0.9$, $\beta_{1}=\beta_{2}=0.5$, $P_{C}=20$, h=0.65, θ=0.7, letting k and s be the independent variables (k∈(20,25], s∈[0,3]), draw graphs of the variation of the decision variables and system members’ profits with k and s, as shown in Figure 5.

From Figure 5, (1) $w_{{}_{1}}^{i},p_{{}_{1}}^{i}(i=F*,L*)$ increase with increases in k, the larger s is, the larger the increase; $q_{{}_{1}}^{i}(i=F*,L*)$ decreases with increases in k, the larger s is, the larger the decrease. It means that the larger k is, the higher the cost required to process remanufactured products, and the members of the supply chain will choose to increase the prices of products to obtain a certain profit. With the increase in carbon emission reduction, the more money the manufacturer invests in low-carbon processes, the greater the price increase will be. At this point, the manufacturer is required to weigh the cost of low-carbon processes, consider the relationship between product prices and demand, and formulate appropriate prices, while the government is required to control the amount of carbon emission reduction by each manufacturer, and also to regulate the price of products to prevent the emergence of sky-high prices. (2) The increase in coefficient of the impact of carbon emission reduction savings on the processing cost of remanufactured products will damage the profits of members of the supply chain system, and the greater the carbon emission reduction, the higher the degree of damage. This requires the manufacturer to consider how to reduce the processing cost of remanufactured products due to enhanced carbon reduction facilities based on accomplishing carbon reduction targets. At the same time, the government can also encourage major supply chain member enterprises to carry out low-carbon process innovation by providing subsidies or opening up green loans.

### 6.2. Discussion

It can be seen from Table 1 that most of the articles related to this paper use the method of game theory to study the different contents. However, most of the existing studies only consider the impact of either the legal constraint recovery rate or the carbon trading mechanism on the supply chain. This paper combines the two to explore their common impact on decision making and profits in the supply chain so as to make the research more realistic and instructive. Vorasayan and Ryan believe that manufacturers in the competitive market can make profits by introducing recycled products [65]. On this basis, this paper defines how the manufacturer can obtain the optimal profit while producing new products and processing remanufactured products at the same time.

The authors of articles [18,20,28] in Section 2.1 all believe that overly strict legal constraints will be detrimental to the profits of the supply chain, while the authors of articles [29,30] believe that strict recovery rates will not always damage the profits of the supply chain. The research conclusions of this paper confirm that the impact of legal constraints on the profits of the supply chain is not absolutely positive or negative. Their relationship depends on the amount of carbon emission reduction and the relevant critical values are also determined in this study. Therefore, the conclusion of this paper has made a breakthrough on the basis of the conclusions of all the above articles.

The authors of articles [34,35,36,37] in Section 2.2 believe that carbon trading policy is better than other carbon policies, which shows that the research on carbon trading policy is valuable. The authors of articles [38,39,40] think that carbon trading price is negatively correlated with the profits of supply chain members, which most people may agree with. However, the research conclusion of this paper disagrees, and the final conclusion is more consistent with article [41]. This paper determines the critical threshold of positive and negative correlation, that is, this paper believes that the relationship between carbon trading price and supply chain profits also depends on the threshold of carbon emission reduction.

The authors of articles [44,45,46] in Section 2.3 think that government intervention is beneficial to the supply chain, but the conclusions of articles [47,48,49] are different. This paper finds through research that the government’s constraint on the manufacturer’s recovery rate and the change of carbon trading price do not have an absolutely positive or negative impact on the supply chain system, that is, based on different constraints, government regulation has a positive or negative impact on the supply chain system. In this paper, the relevant critical conditions are determined, and the above two different research conclusions are combined and improved.

## 7. Conclusions and Managerial Implications

### 7.1. Conclusions

This paper explores the impact of legal recycling constraints and the carbon trading mechanism on the decision making and profitability of members of the supply chain, and the results of this paper show that:

(1) Legal constraints on the minimum recycling rate are necessary. When the carbon reduction per unit of remanufactured product is low, the manufacturer tends to choose a higher recycling rate. Most manufacturer enterprises will then try to achieve the highest recycling rate to maximize profits, and the minimum recycling rate by law can restrain some small and medium-sized manufacturer enterprises whose low-carbon process is not up to standard. When the carbon reduction per unit of remanufactured product is high, the manufacturer needs to pay some price to achieve carbon emission reduction as well as minimize losses. It will thus choose a lower recycling rate and, at this point, it is extremely important to legally regulate the minimum recycling rate, which can not only ensure the recycling of used products to advance steadily but also effectively avoid the phenomenon of manufacturer enterprises avoiding taking social responsibility, which is conducive to the early achievement of carbon emission reduction targets.
(2) Legal constraints are not always conducive to increasing the profits of supply chain system members. When carbon emissions are below a certain threshold, manufacturer enterprises who have the ability to achieve low-carbon production will choose the highest recycling rate to achieve maximum profits, which is a more favorable aspect from the standpoint of achieving carbon reduction goals. At this time the minimum recycling rate of the legal constraint is used to restrain those manufacturer enterprises who avoid undertaking the task of carbon emission reduction. When carbon emissions are above a certain threshold, manufacturer enterprises will choose the lowest recycling rate to achieve maximum profit because the legal constraints at this time will hurt their interests to a certain extent.
(3) Under the carbon trading mechanism, the relationship between carbon trading price and profits of members of the supply chain system depends on the size of carbon reduction per unit of remanufactured product. When the carbon emission reduction amount s is small, the carbon trading price is negatively correlated with the profits of supply chain system members and the smaller the s is, the stronger the negative correlation is; conversely, when the carbon emission reduction amount s is large, the carbon trading price is positively correlated with the profits of supply chain system members and the larger the s is, the stronger the positive correlation is. These indicate that the more perfect the carbon reduction process is, the higher the profit of carbon trading will be, and compared to the traditional mechanism of limiting fixed carbon quotas, the carbon trading mechanism using the grandfathering allocation method can allocate carbon quotas to each manufacturer more flexibly and reasonably, which is more beneficial to environmental benefits.
(4) Achieving carbon emission reduction targets requires policy support from the government. To reach the goal of carbon neutrality, limiting carbon emissions will inevitably harm the interests of some manufacturer enterprises. To motivate them to actively take social responsibility for carbon emission reduction, the government should establish a sound reward and punishment mechanism to reward manufacturers with excellent carbon emission reduction efforts and punish manufacturers with excessive carbon emissions. In addition, it is also hoped that the government can give certain subsidies and policy assistance to manufacturers who have difficulties in carbon emission reduction while planning low-carbon industries rationally, so as to promote the low-carbon transformation of manufacturers with high carbon emissions and accelerate the achievement of carbon emission reduction targets.

### 7.2. Managerial Implications

From the point of view of the manufacturer, as the leader of the supply chain it obtains the highest profit and should take the initiative to assume social responsibilities by selecting environmentally friendly raw materials and using low-carbon production technologies. If the cost consumed by carbon emission reduction efforts is beyond what the manufacturer can afford, it can choose to temporarily stop production if necessary, make profits by selling carbon quotas, and resume production after capital turnover is opened.

From the point of view of government regulation, the government is able to incentivize and guide manufacturers to develop low-carbon industries and produce low-carbon products by rewarding manufacturer enterprises with outstanding carbon reduction efforts, implementing tax subsidies, and offering financing support.

### 7.3. Study Limitations

There are some limitations to this article.

(1) The article study is based on the carbon trading mechanism using the grandfathered allocation method, but in reality many countries and regions also adopt the benchmark carbon quota allocation method. The research in this paper fails to consider the impact of the benchmark quota allocation method on the recycling of used products.
(2) The article assumes that consumers’ perceptions of new and remanufactured products are consistent, but in reality many consumers have dissimilar consumption preferences between new products and remanufactured products. Therefore, if the influence of consumer preferences is considered, a more in-depth study using the article’s model will lead to more conclusions with more realistic guidance value.

Therefore, in future research, considering the impact of the carbon quota allocation mode of the benchmark system on the recycling of waste products, and adding the impact of consumer preferences and other real-world factors, a more in-depth study of the model in this paper will draw more practical conclusions, which is also the next research direction of this article.

## Figures and Tables

**Table 1:** The differences between this paper and other literature.

Literature	Low Carbon Policy	Legal Constraints on Recovery Rates	Waste Recycling	Carbon Trading	Closed-Loop Supply Chain	Game Theory
[18]		√	√			√
[31]		√	√			√
[50]	carbon cap	√	√			√
[30]		√	√		√	√
[48]	carbon subsidy		√		√	√
[52]	carbon tax/subsidy		√		√	√
[40]	cap-and-trade		√	√	√	√
[46]	cap-and-trade			√	√	√
[37]	cap-and-trade			√		√
[38]	cap-and-trade			√		√
[53]	carbon trading			√		√
This paper	carbon trading	√	√	√	√	√

**Table 2:** Variables and parameters involved in the model.

Decision Variables	
$w_{1}$	Wholesale price per unit of new product in the first period, the manufacturer’s decision variable.
$w_{2}$	Wholesale price per unit of product (including new and remanufactured products) in the second period, the manufacturer’s decision variable.
h	The market recovery rate of used products, the manufacturer’s decision variable.
$p_{1}$	Retail price per unit of new product in the first period, the retailer’s decision variable.
$p_{2}$	Retail price per unit of product (including new and remanufactured products) in the second period, the retailer’s decision variable.
**Model parameters**	
Q	Potential maximum market demand per period.
$q_{1}$	Market demand for products in the first period, assuming $q_{1}=Q-\beta_{1}p_{1}$, where $\beta_{1}$ represents the price elasticity coefficient of the first period.
$q_{2}$	Market demand for products in the second period (including new and remanufactured products), assuming $q_{2}=Q-\beta_{2}p_{2}$, where $\beta_{2}$ represents the price elasticity coefficient of the second period.
$c,c_{0}$	Unit production costs of new and remanufactured products, respectively, $0<c<c_{0}$.
$e,e_{0}$	Unit production carbon emissions of new and remanufactured products, respectively, $e>e_{0}$.
θ	Carbon emission reduction coefficient, 0<θ<1.
$P_{C}$	The unit trading price of carbon allowances in the carbon trading market.
$h_{0}$	Minimum recovery rate required by law.
$h_{1}$	The maximum recovery rate of used products, $h_{1}\geq h\geq h_{0}\geq 0$.
μ	The minimum processing cost of remanufactured products, that is, the cost of processing using ordinary processes, and 0<μ<c.
k	Influence coefficient of carbon emission reduction savings $\left(e-e_{0}\right)$ on the processing cost of remanufactured products, and k>0.
$\pi_{m}$	The manufacturer’s profit.
$\pi_{r}$	The retailer’s profit.

**Table 3:** Optimal decisions with the corresponding optimal profits.

Model	Condition	Optimal Recovery Rate	Optimal Decisions and Corresponding Optimal Profits
F-model	$s>s_{1}$	0	$w_{1}^{F*}=\frac{Q}{2\beta_{1}}+g_{2}$, $w_{2}^{F*}=\frac{Q}{2\beta_{2}}+g_{1}$, $p_{1}^{F*}=\frac{3Q}{4\beta_{1}}+\frac{g_{2}}{2}$, $p_{2}^{F*}=\frac{3Q}{4\beta_{2}}+\frac{g_{1}}{2}$, $q_{1}^{F*}=\frac{Q-2g_{2}\beta_{1}}{4}$, $q_{2}^{F*}=\frac{Q-2g_{1}\beta_{2}}{4}$, $\pi_{r}^{F*}=\frac{Q^{2}\beta }{16\beta_{1}\beta_{2}}+\frac{\beta_{1}g_{2}^{2}+\beta_{2}g_{1}^{2}-gQ}{4}$ $\pi_{m}^{F*}=\frac{Q^{2}\beta }{8\beta_{1}\beta_{2}}+\frac{\beta_{1}g_{2}^{2}+\beta_{2}g_{1}^{2}-gQ}{2}$, $\pi^{F*}=\pi_{m}^{F*}+\pi_{r}^{F*}$.
	$s<s_{1}$	$h_{1}$	$w_{1}^{F*}=\frac{Q+(s-s_{1})h_{1}\delta \beta_{1}}{2\beta_{1}}+g_{2}$, $w_{2}^{F*}=\frac{Q}{2\beta_{2}}+g_{1}$, $p_{1}^{F*}=\frac{3Q}{4\beta_{1}}+\frac{g_{2}}{2}+\frac{(s-s_{1})\delta h_{1}}{4}$, $p_{2}^{F*}=\frac{3Q}{4\beta_{2}}+\frac{g_{1}}{2}$, $q_{1}^{F*}=\frac{Q-(s-s_{1})\delta h_{1}\beta_{1}}{4}-\frac{g_{2}\beta_{1}}{2}$, $q_{2}^{F*}=\frac{Q-2g_{1}\beta_{2}}{4}$ $\pi_{r}^{F*}=\frac{\beta_{2}g_{1}^{2}-Qg}{4}+\frac{Q^{2}\beta }{16\beta_{1}\beta_{2}}+\frac{\beta_{1}{\left[2g_{2}+(s-s_{1})h_{1}\delta \right]}^{2}}{16}-\frac{(s-s_{1})Q\delta h_{1}}{8}$$\pi_{m}^{F*}=\frac{\beta_{2}g_{1}^{2}-Qg}{2}+\frac{Q^{2}\beta }{8\beta_{1}\beta_{2}}+\frac{\beta_{1}{\left[2g_{2}+h_{1}\delta (s-s_{1})\right]}^{2}}{8}-\frac{Qh_{1}\delta (s-s_{1})}{4}$, $\pi^{F*}=\pi_{m}^{F*}+\pi_{r}^{F*}$.
L-model	$s>s_{1}$	$h_{0}$	$w_{1}^{L*}=\frac{Q+(s-s_{1})h_{0}\delta \beta_{1}}{2\beta_{1}}+g_{2}$, $w_{2}^{L*}=\frac{Q_{2}}{2\beta_{2}}+g_{1}$, $p_{1}^{L*}=\frac{3Q}{4\beta_{1}}+\frac{g_{2}}{2}+\frac{(s-s_{1})\delta h_{0}}{4}$, $p_{2}^{F*}=\frac{3Q}{4\beta_{2}}+\frac{g_{1}}{2}$, $q_{1}^{L*}=\frac{Q-(s-s_{1})\delta h_{0}\beta_{1}}{4}-\frac{g_{2}\beta_{1}}{2}$, $q_{2}^{F*}=\frac{Q-2g_{1}\beta_{2}}{4}$, $\pi_{r}^{L*}=\frac{\beta_{2}g_{1}^{2}-Qg}{4}+\frac{Q^{2}\beta }{16\beta_{1}\beta_{2}}+\frac{\beta_{1}{\left[2g_{2}+(s-s_{1})h_{0}\delta \right]}^{2}}{16}-\frac{(s-s_{1})Q\delta h_{0}}{8}$, $\pi_{m}^{L*}=\frac{\beta_{2}g_{1}^{2}-Qg}{2}+\frac{Q^{2}\beta }{8\beta_{1}\beta_{2}}+\frac{\beta_{1}{\left[2g_{2}+(s-s_{1})h_{0}\delta \right]}^{2}}{8}-\frac{(s-s_{1})Q\delta h_{0}}{4}$, $\pi^{L*}=\pi_{m}^{L*}+\pi_{r}^{L*}$.
	$s<s_{1}$	$h_{1}$	The optimal decision is the same as the F-model when $s<s_{1}$.
Remarks			$c_{0}=\mu +k(e-e_{0})$, $\beta =\beta_{1}+\beta_{2}$, $\delta =k-P_{C}$, $s=e-e_{0}$, $s_{1}=\frac{c-\mu }{k-P_{C}}$$(k-P_{C}>0$, c−μ>0, i.e.; $s_{1}\neq 0)$, $g_{1}=\frac{c+eP_{C}}{2}$, $g_{2}=\frac{c-eP_{C}\theta }{2}$, $g=g_{1}+g_{2}$.

**Table 4:** Comparison of optimal decisions and optimal profits of two models.

Condition	Optimal Recovery Rate	Optimal Decisions and Corresponding Optimal Profits
$s>s_{1}$	$h^{F*}=0$ $h^{L*}=h_{0}$	$w_{1}^{F*}<w_{1}^{L*}$, $w_{2}^{F*}=w_{2}^{L*}$, $p_{1}^{F*}<p_{1}^{L*}$, $p_{2}^{F*}=p_{2}^{L*}$, $q_{1}^{F*}>q_{1}^{L*}$, $q_{2}^{F*}=q_{2}^{L*}$, $\pi_{m}^{F*}>\pi_{m}^{L*}$, $\pi_{r}^{F*}>\pi_{r}^{L*}$, $\pi^{F*}>\pi^{L*}$
$s<s_{1}$	$h^{F*}=h^{L*}=h_{1}$	$w_{1}^{F*}=w_{1}^{L*}$, $w_{2}^{F*}=w_{2}^{L*}$, $p_{1}^{F*}=p_{1}^{L*}$, $p_{2}^{F*}=p_{2}^{L*}$, $q_{1}^{F*}=q_{1}^{L*}$, $q_{2}^{F*}=q_{2}^{L*}$, $\pi_{m}^{F*}=\pi_{m}^{L*}$, $\pi_{r}^{F*}=\pi_{r}^{L*}$, $\pi^{F*}=\pi^{L*}$

## Data Availability

No new data were created or analyzed in this study. Data sharing is not applicable to this article.

## Appendix A

Since $hq_{1}<q_{2}$, the maximum recycling rate of used products cannot reach $\frac{q_{2}}{q_{1}}$, so the recycling rate of used products determined by the manufacturer must satisfy $h_{0}\leq h\leq h_{1}<\frac{q_{2}}{q_{1}}$. According to Equation (2), it can be known from $\frac{\partial^{2}\pi_{r}}{\partial p_{1}{}^{2}}<0,\frac{\partial^{2}\pi_{r}}{\partial p_{2}{}^{2}}<0$ that $\pi_{r}$ is a concave function with respect to $p_{1}$ and $p_{2}$. The reaction functions of $\pi_{r}$ with respect to $p_{1}$ and $p_{2}$ are:

(A1) $$p_{1}=\frac{Q+w_{1}\beta_{1}}{2\beta_{1}},p_{2}=\frac{Q+w_{2}\beta_{2}}{2\beta_{2}}$$

The corresponding reaction functions for the first and second cycle market demand are:

(A2) $$q_{1}=\frac{Q-w_{1}\beta_{1}}{2},q_{2}=\frac{Q-w_{2}\beta_{2}}{2}$$

Substitute (A1) and (A2) into Equation (1) and apply the Kuhn–Tucker condition to discuss the optimal solution of the model.

(A) The solution process for the F-model without legal recycling constraints is as follows:

Constructing the manufacturer’s Lagrangian function L, let $L=\pi_{m}+\lambda_{1}h-\lambda_{2}\left(h-h_{1}\right)$, the resulting decision variables need to satisfy:

(A3) $$\frac{\partial L}{\partial w_{1}}=0,\frac{\partial L}{\partial w_{2}}=0,\frac{\partial L}{\partial h}=0,\lambda_{1}h=0,\lambda_{2}(h-h_{1})=0$$

According to condition (A3), it is obtained that:

1. When $\lambda_{1}=\lambda_{2}=0$, the solution can be obtained as:
  $$\begin{array}{l} w_{1}^{*}=\frac{Q}{\beta_{1}},\\ w_{2}^{*}=\frac{Q+c\beta_{2}+eP_{C}\beta_{2}}{2\beta_{2}}\\ h=\frac{Q-c\beta_{1}+eP_{C}\beta_{1}\theta }{\beta_{1}(ek-c-e_{0}k-eP_{C}+e_{0}P_{C}+\mu )} \end{array}$$

From $w_{1}^{*}=\frac{Q}{\beta_{1}}$, the market price $p_{1}=\frac{Q}{\beta_{1}}$ of the retailer in the first cycle and the market demand in the first period is $q_{1}=Q-\beta_{1}p_{1}=0$, which goes against reality so this solution is therefore discarded.

2. When $\lambda_{1}>0,\lambda_{2}=0$, the solution can be obtained as:
  $$\begin{array}{l} w_{1}^{*}=\frac{Q+c\beta_{1}-e\theta P_{C}\beta_{1}}{2\beta_{1}}\\ w_{2}^{*}=\frac{Q+c\beta_{2}+eP_{C}\beta_{2}}{2\beta_{2}}\\ h=0\\ \lambda_{2}=\frac{1}{4}[c-\mu -(e-e_{0})(k-P_{C})][(c-e\theta P_{C})\beta_{1}-Q] \end{array}$$

From the above expressions, it can be seen that all the decision variables are greater than 0, so it is only necessary to satisfy $\lambda_{2}=\frac{1}{4}[c-\mu -(e-e_{0})(k-P_{C})][(c-e\theta P_{C})\beta_{1}-Q]>0$. Obviously, $(c-e\theta P_{C})\beta -Q_{1}<0$ holds constant, so only $c-\mu -(e-e_{0})(k-P_{C})<0$ is required, that is, $e-e_{0}>\frac{c-\mu }{k-P_{C}}=s_{1}$ (which necessarily has $k-P_{C}>0$, if $k-P_{C}<0$, then the carbon emission reduction $e-e_{0}<0$, contradictory to the question). At this point, the optimal solution is obtained at $e-e_{0}>s_{1}$.

3. When $\lambda_{1}=0,\lambda_{2}>0$, the solution can be obtained as:
  $$\begin{array}{l} w_{1}^{*}=\frac{Q+\beta_{1}[c-ch_{1}+e(h_{1}k-h_{1}P_{C}-P_{C}\theta )+h_{1}(-e_{0}k+e_{0}P_{C}+\mu )]}{2\beta_{1}}\\ w_{2}^{*}=\frac{Q+c\beta_{2}+eP_{C}\beta_{2}}{2\beta_{2}}\\ h=h_{1}\\ \lambda_{2}=\frac{1}{4}[c-\mu -(e-e_{0})(k-P_{C})][Q+\beta_{1}(ch_{1}-c-eh_{1}k+e_{0}h_{1}k+eh_{1}P_{C}-e_{0}h_{1}P_{C}+eP_{C}\theta -h_{1}\mu )] \end{array}$$

From the above expressions, it can be seen that all the decision variables are greater than 0, so it is only necessary to satisfy the parameter expression $\lambda_{2}>0$, where obviously $Q+\beta_{1}(ch_{1}-c-eh_{1}k+e_{0}h_{1}k+eh_{1}P_{C}-e_{0}h_{1}P_{C}+eP_{C}\theta -h_{1}\mu )>0$ holds constant, so only $c-\mu -(e-e_{0})(k-P_{C})>0$ is required, that is, $e-e_{0}<\frac{c-\mu }{k-P_{C}}=s_{1}$ (which necessarily has $k-P_{C}>0$). At this point, the optimal solution is obtained at $e-e_{0}<s_{1}$.

4. When $\lambda_{1}>0,\lambda_{2}>0$, it is obtained from $\lambda_{1}\left(h_{0}-h\right)=0,\lambda_{2}\left(h-h_{1}\right)=0$ that $h=h_{0}=h_{1}$. The minimum recovery rate is equal to the maximum recovery rate, so the relevant discussion at this time is meaningless.

To sum up, when $e-e_{0}<s_{1}$ or $e-e_{0}>s_{1}$ holds, the optimal solution exists.

Bringing the obtained wholesale price into (A1), we can find the optimal retail price, which in turn can be solved for other optimal decisions, as well as the optimal profit of members of the supply chain system.

(B) The solution process for the L-model with legal recovery constraints is the same as the F-model. Due to space limitations, it is omitted here.

## Appendix B

**Proof of Proposition** **1.** When $s>s_{1}$, both the F-model and L-model satisfy:

$$\begin{array}{l} \frac{\partial w_{1}^{i}}{\partial k}=\frac{(e-e_{0})h}{2},\frac{\partial w_{2}^{i}}{\partial k}=0,\frac{\partial p_{1}^{i}}{\partial k}=\frac{(e-e_{0})h}{4},\frac{\partial p_{2}^{i}}{\partial k}=0,\frac{\partial q_{1}^{i}}{\partial k}=-\frac{(e-e_{0})h\beta_{1}}{4},\frac{\partial q_{2}^{i}}{\partial k}=0\\ \frac{\partial w_{1}^{i}}{\partial e_{0}}=\frac{h(P_{C}-k)}{2},\frac{\partial w_{2}^{i}}{\partial e_{0}}=0,\frac{\partial p_{1}^{i}}{\partial e_{0}}=\frac{h(P_{C}-k)}{4},\frac{\partial p_{2}^{i}}{\partial e_{0}}=0,\frac{\partial q_{1}^{i}}{\partial e_{0}}=\frac{h(k-P_{C})\beta_{1}}{4},\frac{\partial q_{2}^{i}}{\partial e_{0}}=0(i=F,L) \end{array}$$

That is, the magnitude of each partial derivative is:

$$\begin{array}{l} \frac{\partial w_{1}^{i}}{\partial k}\geq 0,\frac{\partial w_{2}^{i}}{\partial k}=0,\frac{\partial p_{1}^{i}}{\partial k}\geq 0,\frac{\partial p_{2}^{i}}{\partial k}=0,\frac{\partial q_{1}^{i}}{\partial k}\leq 0,\frac{\partial q_{2}^{i}}{\partial k}=0\\ \frac{\partial w_{1}^{i}}{\partial e_{0}}\leq 0,\frac{\partial w_{2}^{i}}{\partial e_{0}}=0,\frac{\partial p_{1}^{i}}{\partial e_{0}}\leq 0,\frac{\partial p_{2}^{i}}{\partial e_{0}}=0,\frac{\partial q_{1}^{i}}{\partial e_{0}}\geq 0,\frac{\partial q_{2}^{i}}{\partial e_{0}}=0(i=F,L) \end{array}$$

Thus $w_{1}^{i},p_{1}^{i}(i=F,L)$ are positively correlated with k and negatively correlated with $e_{0}$; $q_{1}^{i}(i=F,L)$ is negatively correlated with k and positively correlated with $e_{0}$; $w_{2}^{i},p_{2}^{i},q_{2}^{i}(i=F,L)$ are not influenced by k or $e_{0}$.

Since:

$$\frac{\partial \pi_{m}^{i}}{\partial k}=\frac{-hsQ+hs[c-h(c-ks-\mu )-(hs+e\theta )P_{C}]\beta_{1}}{4}\leq 0$$

$$\frac{\partial \pi_{r}^{i}}{\partial k}=\frac{-hsQ+hs[c-h(c-ks-\mu )-(hs+e\theta )P_{C}]\beta_{1}}{8}\leq 0$$

$$\frac{\partial \pi^{i}}{\partial k}=\frac{-3hsQ+3hs[c-h(c-ks-\mu )-(hs+e\theta )P_{C}]\beta_{1}}{8}\leq 0$$

the same can be proven that: $\frac{\partial \pi_{m}^{i}}{\partial e_{0}}\geq 0$, $\frac{\partial \pi_{r}^{i}}{\partial e_{0}}\geq 0$, $\frac{\partial \pi^{i}}{\partial e_{0}}\geq 0$.

In all the above equations, i=F,L, $s=e-e_{0}$, $s_{1}=\frac{c-\mu }{k-P_{C}}$, so when $s>s_{1}$, $\pi_{m}^{i},\pi_{r}^{i},\pi^{i},(i=F,L)$ are negatively correlated with k and positively correlated with $e_{0}$. Similarly, it can be proven that the conclusions also hold and are strictly valid when $s<s_{1}$. □
